# Can We Use Satellite-Based FAPAR to Detect Drought?

**DOI:** 10.3390/s19173662

**Published:** 2019-08-23

**Authors:** Jian Peng, Jan-Peter Muller, Simon Blessing, Ralf Giering, Olaf Danne, Nadine Gobron, Said Kharbouche, Ralf Ludwig, Ben Müller, Guoyong Leng, Qinglong You, Zheng Duan, Simon Dadson

**Affiliations:** 1 School of Geography and the Environment, University of Oxford, Oxford OX1 3QY, UK; 2 Department of Geography, University of Munich (LMU), 80333 Munich, Germany; 3 Institute for Climate and Global Change Research, School of Atmospheric Sciences, Nanjing University, Nanjing 210023, China; 4 Imaging Group, Mullard Space Sciences Laboratory, University College London, Department of Space and Climate Physics, Holmbury, St Mary RH5 6NT, UK; 5 FastOpt GmbH, Schanzenstraße 36, D-20357 Hamburg, Germany; 6 Brockmann Consult GmbH, Max-Plack Str.2, 21502 Geesthacht, Germany; 7 European Commission, Joint Research Centre, Via Enrico Fermi 2749, 21027 Ispra, Italy; 8 Key Laboratory of Water Cycle and Related Land Surface Processes, Institute of Geographic Sciences and Natural Resources Research, Chinese Academy of Sciences, Beijing 100101, China; 9 Environmental Change Institute, University of Oxford, Oxford OX1 3QY, UK; 10 Department of Atmospheric and Oceanic Sciences & Institute of Atmospheric Sciences, Fudan University, Shanghai 200438, China; 11 Department of Physical Geography and Ecosystem Science, Lund University, S-223 62 Lund, Sweden; 12 Lancaster Environment Centre, Lancaster University, Lancaster LA1 4YQ, UK

**Keywords:** FAPAR, QA4ECV, drought, MODIS, CGLS, Australia

## Abstract

Drought in Australia has widespread impacts on agriculture and ecosystems. Satellite-based Fraction of Absorbed Photosynthetically Active Radiation (FAPAR) has great potential to monitor and assess drought impacts on vegetation greenness and health. Various FAPAR products based on satellite observations have been generated and made available to the public. However, differences remain among these datasets due to different retrieval methodologies and assumptions. The Quality Assurance for Essential Climate Variables (QA4ECV) project recently developed a quality assurance framework to provide understandable and traceable quality information for Essential Climate Variables (ECVs). The QA4ECV FAPAR is one of these ECVs. The aim of this study is to investigate the capability of QA4ECV FAPAR for drought monitoring in Australia. Through spatial and temporal comparison and correlation analysis with widely used Moderate Resolution Imaging Spectroradiometer (MODIS), Satellite Pour l’Observation de la Terre (SPOT)/PROBA-V FAPAR generated by Copernicus Global Land Service (CGLS), and the Standardized Precipitation Evapotranspiration Index (SPEI) drought index, as well as the European Space Agency’s Climate Change Initiative (ESA CCI) soil moisture, the study shows that the QA4ECV FAPAR can support agricultural drought monitoring and assessment in Australia. The traceable and reliable uncertainties associated with the QA4ECV FAPAR provide valuable information for applications that use the QA4ECV FAPAR dataset in the future.

## 1. Introduction

Hydroclimatic extremes such as heat waves and droughts are a common occurrence in arid and semiarid areas of the world such as Australia [1]. These extreme events generally have significant impacts on humans, the environment, and the economy [2]. Specifically, most of Australia has experienced severe droughts and heat waves in recent decades, which have induced significant stress on natural environmental and socioeconomic systems [3,4]. For example, the “Millennium Drought” (2001–2009) in southeast Australia caused severe damage to ecosystems and agriculture, and led to the enforcement of water restrictions in many cities [5,6]. The 2009 Victorian heat wave killed more than 370 people with insured losses of 1.3 billion US dollars [4]. Climate projections show that there will be a further increase in the frequency and severity of hydroclimatic extreme events in Australia [7]. Therefore, it is important to monitor and investigate the occurrence of droughts in Australia.

Generally, droughts and their associated impacts are difficult to define and can be classified as meteorological, hydrological, agricultural, environmental, and socioeconomic based on different drought characteristics of duration, intensity, and spatial and temporal extent [8,9,10]. Traditionally, point-based in-situ measurements of hydrometeorological variables such as precipitation, soil moisture, temperature, and streamflow have been used to track the severity and location of droughts [11,12,13]. However, it is very challenging to monitor spatial and temporal variability of drought with point observations from in-situ instruments. In addition, there are many areas where hydrometeorological observation networks are not established [14]. Satellite remote sensing provides a valuable way to monitor drought operationally over large areas. Compared with in-situ ones, satellite-based measurements have the advantages of global long-term observations, multiple spatial resolutions, and consistent data records [15,16]. Remote sensing observations from optical, thermal, and microwave bands have been used to retrieve drought-related variables including precipitation, soil moisture, evapotranspiration, snow cover, land surface temperature, vegetation, and total water storage over recent decades [17,18,19,20]. Hydrological variables such as precipitation, soil moisture, and evapotranspiration can be converted to drought indicators such as Standardized Precipitation Index (SPI), Standardized Precipitation Evapotranspiration Index (SPEI), and Evaporative Stress Index (ESI) [21,22,23,24]. These indicators can be used to categorize and quantify drought extent and severity [15,25]. Satellite observations can also be used to monitor and assess the impacts of drought on the ecosystem. The optical and thermal band satellite observations have been widely used to monitor vegetation changes and water stress of plants [26,27].

The most commonly used vegetation index for monitoring agricultural drought is the Normalized Difference Vegetation Index (NDVI) [28]. There are also a number of other indices based on optical and thermal bands such as the Vegetation Condition Index (VCI), Temperature Condition Index (TCI), Normalized Difference Water Index (NDWI), and Vegetation Health Index (VHI) [15,29]. The idea behind analyzing these indices is that rainfall stress can result in photosynthetic capacity reduction of vegetation and changes in absorbed photosynthetic radiation by plants [30]. In addition to these indices, the Fraction of Absorbed Photosynthetically Active Radiation (FAPAR) can directly reflect the greenness and health conditions of vegetation [31,32]. A number of studies have used FAPAR to monitor and assess drought impacts [33,34,35]. In particular, FAPAR has been selected and operationally used as the Combined Drought Indicator (CDI) for monitoring drought in the European Drought Observatory (EDO) within the Copernicus Emergency Management Service [30].

At present, several satellite-based global FAPAR datasets have been produced and updated routinely for various applications. These FAPAR datasets include the Moderate Resolution Imaging Spectroradiometer (MODIS), Satellite Pour l’Observation de la Terre VEGETATION (SPOT-VEGETATION), Advanced Very High Resolution Radiometer (AVHRR), Sentinel-3, and Joint Research Center (JRC) FAPAR. Although these FAPAR products have been widely validated and applied to a number of applications including drought monitoring, disagreements and inconsistencies amongst these datasets have been reported by several studies [36,37]. The discrepancies between these datasets are mainly due to different retrieval methods, assumptions, and definitions of FAPAR [38]. Therefore, as identified by Global Climate Observing System (GCOS) Implementation Plan (IP), a quality-assured long-term FAPAR dataset is urgently required, which can provide reliable and traceable quality information. With this background, the Quality Assurance for Essential Climate Variables (QA4ECV) project developed a quality assurance framework to provide understandable and traceable quality information for Essential Climate Variables (ECVs) (http://www.qa4ecv.eu) [39,40]. Within the QA4ECV project, long-term and quality assured FAPAR datasets were recently released. These QA4ECV FAPAR datasets have great potential for drought monitoring and assessment. The main objective of this study is a comprehensive investigation of the performance of the QA4ECV FAPAR for drought monitoring over Australia. After introducing the study region in Section 2, a detailed introduction to the QA4ECV FAPAR dataset, other FAPAR datasets, soil moisture, and the SPEI drought index are described in Section 3. The fourth section discusses and evaluates the results. A concluding section summarizes the results of the whole study.

## 2. Study Area

**Figure 1:**
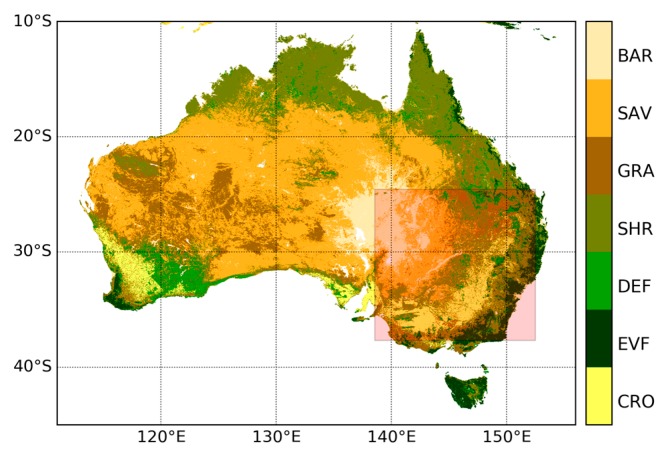
European Space Agency Climate Change Initiative (ESA CCI) land cover type map for Australia divided into bare areas (BAR), savannas (SAV), grasslands (GRS), shrublands (SHR), deciduous forest (DEF), evergreen forest (EVF), and croplands (CRO). The area with light red color denotes the extent of Murray–Darling Basin.

The current study was performed over Australia, which is the most drought-prone inhabited continent in the world. Almost 70% of the whole continent is arid or semiarid land. Only the southwestern and southeastern parts have moderately fertile soil and temperate climates. The northern part of Australia has a tropical monsoonal climate. Australia has an annual average rainfall of 499 mm and annual runoff of 70 mm, indicating only 14% of rainfall is runoff [41]. Owing to the influences of climate variability modes of the El Niño-Southern Oscillation (ENSO) and Indian Ocean Dipole (IOD), the rainfall over Australia varies significantly from year to year, further inducing regular drought cycles [42]. These climatic characteristics make Australia one of the countries most affected by extensive droughts. The seasonal dynamics of vegetation across Australia also present significant variations due to the complex climate and surface conditions [43]. Figure 1 shows the land cover map generated from the European Space Agency (ESA) Climate Change Initiative (CCI) land cover dataset for the year 2010 [44,45]. It can be seen that savannah, grassland, and bare areas dominate the majority of the continent, whilst forests are mainly located in coastal areas.

## 3. Data and Methods

### 3.1. Satellite-Based FAPAR Datasets

#### 3.1.1. QA4ECV FAPAR

The QA4ECV project produced two FAPAR datasets. One was created from a long albedo time series measured using the Advanced Very High Resolution Radiometer (AVHRR) combined with geostationary instruments using the 5D Two-stream Inversion Package (TIP, referred as BHR-TIP FAPAR) [40]. This represents the diffuse component of the FAPAR. Another FAPAR was generated from daily spectral measurements acquired by AVHRR on board a series of National Oceanic and Atmospheric Administration (NOAA) platforms using the JRC algorithm for the retrieval of Directional Hemispherical Reflectance FAPAR [46]. This is known as the green and direct FAPAR. Specifically, the BHR-TIP FAPAR was derived with the TIP retrieval method from white-sky albedos (BHR) in the visible and near-infrared broadband [47]. TIP delivers a Gaussian approximation of the probability density functions (PDFs) of the retrieved model parameters of a 1D canopy model which characterizes the radiative status of the vegetation–soil system. The method aims at consistency with large-scale climate and Earth system models and does not require assumptions about other factors (e.g., biome type) to be made. In general, the TIP-FAPAR based on various albedo products has been widely validated, such as shown in [47,48,49]. Verification studies within the QA4ECV project revealed that a bias in the QA4ECV albedo product introduces noise and non-systematic biases into the BHR-TIP FAPAR product, which are expected to be present in the monthly product (http://www.qa4ecv.eu/sites/default/files/D5.4_v1.0.pdf). A reprocessing of the QA4ECV albedo dataset is planned. Based on the global daily and monthly albedo data on 0.5 degree and 0.05 degree regular grids, BHR-TIP FAPAR is available for 35 years, from 1982 to 2016 (http://www.qa4ecv.eu/ecv/laifapar-p). In this study, the BHR-TIP FAPAR was used due to its longer temporal coverage than Directional Hemispherical Reflectance (DHR) FAPAR. Therefore, the QA4ECV FAPAR further refers to BHR-TIP FAPAR in this paper.

#### 3.1.2. MODIS FAPAR

The MODIS FAPAR was calculated from the atmospherically corrected surface reflectance observed by MODIS installed on the NASA Terra and Aqua satellites. The main retrieval method is based on three-dimensional radiative transfer model inversion, from which the clumping at canopy scale is accounted [50]. The radiative transfer model inversion is realized with a Look-Up-Table (LUT), which is separated into 8 biome types to represent structurally different three-dimensional vegetation canopy types [47]. If the main radiative transfer retrieval method fails, an empirical method based on the relationship between the NDVI and FAPAR is used to retrieve FAPAR [50]. A detailed description of the radiative transfer method and empirical algorithm can be found in the Algorithm Theoretical Basis Document (ATBD) [51]. The MODIS Collection 6 FAPAR dataset has a temporal resolution of eight days and spatial resolution of 500 m, which has been comprehensively evaluated with ground-based measurements and inter-compared with other FAPAR products by many studies such as [52,53].

#### 3.1.3. SPOT/PROBA-V FAPAR

The SPOT/PROBA-V FAPAR dataset was generated from SPOT VEGETATION (1998–2014) and Project for On-Board Autonomy-Vegetation (PROBA-V) mission (2013–now) observations [54]. The retrieval algorithm is based on fusing and scaling MODIS and CYCLOPES FAPAR products via a neural network approach to obtain the ‘best estimate’ of FAPAR. The satellite-observed top of canopy directional normalized reflectance serves as input data. Detailed information on the retrieval method can be found in [54]. The SPOT/PROBA-V FAPAR dataset is delivered on a 1/112° grid every 10 days. The dataset has been validated for different biomes and reported to have good quality by many studies such as [55,56]. The version 2 of this dataset provided by COPERNICUS Global Land Service (CGLS) is used in the current study. In the following parts, CGLS FAPAR is referred to SPOT/PROBA-V FAPAR.

### 3.2. ESA CCI Soil Moisture

The European Space Agency’s Climate Change Initiative (ESA CCI) soil moisture product is an unique multi-decadal soil moisture record, which was generated by merging several microwave satellite soil moisture products together into harmonized datasets [57,58]. The ESA CCI soil moisture has a spatial resolution of 0.25° and daily temporal resolution, covering the period from 1978 to 2018 (v04.2). The ESA CCI soil moisture comprises three products, namely active, passive, and combined soil moisture datasets. The accuracy of the products has been carefully evaluated with in-situ measurements by many studies such as [59,60,61]. Furthermore, the products have been successfully applied for various applications such as drought analysis [62], climate model evaluation [63], and hydrological monitoring and prediction [64]. A recent review paper comprehensively summarizes the accuracy, applications, and future algorithms of the ESA CCI soil moisture [65].

### 3.3. SPEI Drought Index

The Standardized Precipitation Evapotranspiration Index (SPEI) is designed to take into account both precipitation and potential evapotranspiration (PET) for determining drought. It is a multi-scalar drought index and can capture the impact of temperature increase on water demand [22]. A global SPEI dataset is available based on Climatic Research Unit (CRU) TS 3.24 input data for the period from 1901 to 2015 [66]. This SPEI dataset is delivered with spatial resolution of 0.5° and monthly temporal resolution. In recent years, this SPEI dataset has been widely used for a number of drought-related studies such as drought monitoring and investigating the response of vegetation to drought [67,68]. It should be noted here that the SPEI used in this study is at a three month scale because the optimal time-scale for integration of SPEI for monitoring agricultural drought was found to be three months [30].

### 3.4. Methods

#### 3.4.1. Data Pre-Processing

All of the datasets used in this study were aggregated to monthly mean values for the period of 2001–2015, which correspond to the common temporal span for all datasets. All the datasets were further aggregated to the same spatial resolution of 0.5°. After that, the standardized anomaly for all FAPAR datasets and soil moisture were calculated during the available temporal extent.

#### 3.4.2. Evaluation Strategies

The characteristics of all the FAPAR datasets were investigated through direct spatial and temporal comparison between products. The spatial patterns of the monthly mean FAPAR were analyzed, including the identification of low and high values. To evaluate the feasibility of FAPAR for drought analysis, the relationship between FAPAR, SPEI, and soil moisture was explored spatially and temporally. To facilitate direct comparison between FAPAR and SPEI as well as soil moisture, both FAPAR and soil moisture are standardized with the following equation:

(1) $$Y=\frac{X_{i}-\bar{X}}{\sigma }$$

where *Y* is the standardized anomaly of FAPAR or soil moisture, *X_i_* is FAPAR or soil moisture for month *i*, $\bar{X}$ and σ are the monthly mean and standard deviation of X for the years from 2001 to 2015. The standardized method has been recommended by many studies for evaluating drought indices such as [69,70]. The correlation between FAPAR, SPEI, and soil moisture is quantified with Spearman’s correlation coefficient (R). In addition, the spatial and temporal variations of all datasets in a typical drought year 2015 were also investigated in order to show the performance of FAPAR for drought detection.

## 4. Results

### 4.1. Comparison of Mean FAPAR Estimates

**Figure 2:**
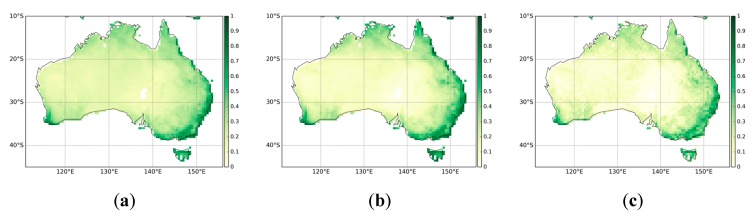
Spatial patterns of monthly mean average Fraction of Absorbed Photosynthetically Active Radiation (FAPAR) for the years 2001–2015: (**a**) Moderate Resolution Imaging Spectroradiometer (MODIS), (**b**) COPERNICUS Global Land Service (CGLS), (**c**) Quality Assurance for Essential Climate Variables (QA4ECV).

**Figure 3:**
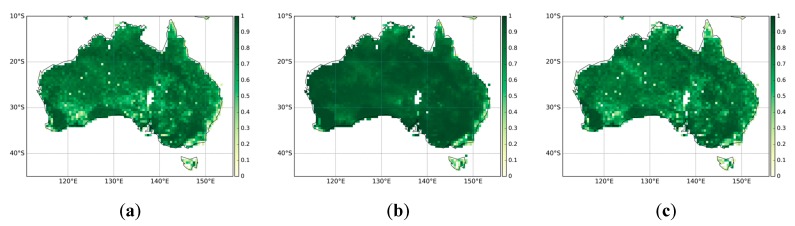
The Pearson correlation coefficient among the FAPAR datasets for the years 2001–2015: (**a**) MODIS against QA4ECV, (**b**) MODIS against CGLS, (**c**) QA4ECV against CGLS.

**Figure 4:**
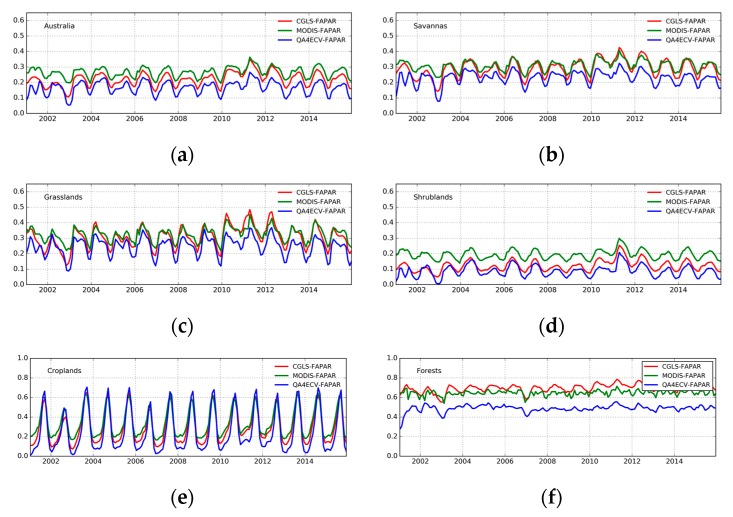
Time series of different FAPAR products over Australia and different land cover types: (**a**) Australia, (**b**) Savannas, (**c**) Grasslands, (**d**) Shrublands, (**e**) Croplands, (**f)** Forests.

Figure 2 presents the monthly mean FAPAR estimates from MODIS, CGLS, and QA4ECV respectively. In general, all three FAPAR show similar patterns with low FAPAR (less than 0.4) present over most parts of the continent. The coastal areas, particularly in the west and east, display high FAPAR values. These patterns are in agreement with the land cover map of Australia (Figure 1). In addition, the Pearson correlation coefficient values between the three FAPAR datasets are shown in Figure 3. It can be seen that MODIS, CGLS, and QA4ECV have good correlation with r values higher than 0.5 for most areas. It is noted that the *p* value is less than 0.05 for all grid cells, which shows that the comparison is statistically significant. Slightly better agreement between MODIS and CGLS can be observed compared to that between MODIS and QA4ECV, CGLS, and QA4ECV. This is because the generation of CGLS FAPAR involves the fusing and scaling of MODIS FAPAR and CYCLOPES FAPAR products. In addition, the time series of the three datasets for Australia and the different land cover types are presented in Figure 4. It can be seen that all datasets show agreement in seasonal variability, while the magnitudes are different among the datasets. Generally high agreement among the datasets occurs for croplands and grasslands, and high disagreement occurs for forests. Slightly better agreement between MODIS and CGLS is found over savannas, while better agreement between QA4ECV and CGLS happens over shrublands. These results are consistent with previous studies such as [36,71], which also found high consistency between different FAPAR products for croplands and substantial disagreement for forests. The reasons for discrepancies among datasets might be different retrieval methods, model-specific assumptions, use of different land cover, and others. In order to quantify differences and validate these FAPAR datasets, more intensive and continuous in-situ measurements over different land cover types are needed. Nevertheless, the generally good spatial correlation and consistent seasonal variability among different FAPAR datasets suggests the potential of a long-term QA4ECV FAPAR dataset for drought monitoring.

### 4.2. Correlation Analysis between FAPAR and SPEI, and Soil Moisture

**Figure 5:**
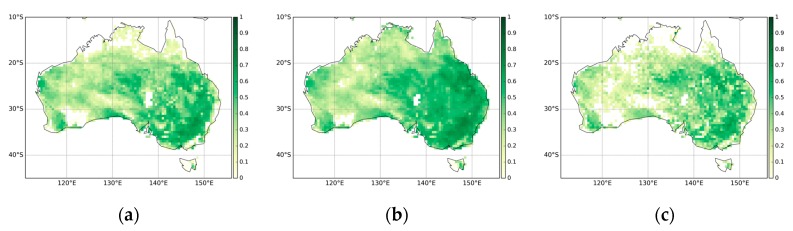
The Pearson correlation coefficient between FAPAR and Standardized Precipitation Evapotranspiration Index (SPEI) during the years 2001–2015: (**a**) MODIS, (**b**) CGLS, (**c**) QA4ECV.

**Figure 6:**
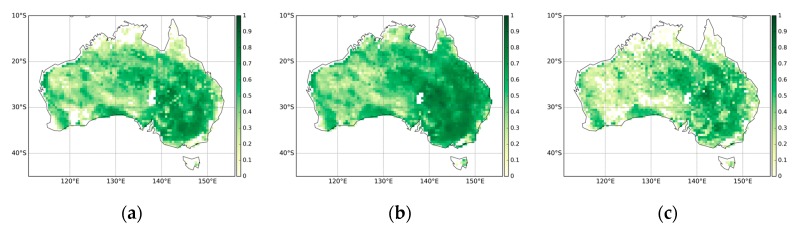
The Pearson correlation coefficient between FAPAR and Climate Change Initiative (CCI) soil moisture during the years 2001–2015: (**a**) MODIS, (**b**) CGLS, (**c**) QA4ECV.

Since SPEI has been widely used for drought monitoring [72,73], the relationship between SPEI and the FAPAR standardized anomaly was explored to test if FAPAR can capture drought signals. Figure 5 shows the r values between different FAPAR products and SPEI during 2001–2015. It can be seen that FAPAR and SPEI generally agree with positive correlations. Better results are shown for CGLS FAPAR (R_mean_ = 0.44) compared to MODIS (R_mean_ = 0.37) and QA4ECV (R_mean_ = 0.35). In terms of spatial areas, all the FAPAR present better correlation with SPEI over eastern Australia than western Australia. In addition, the correlation between FAPAR and CCI soil moisture was also calculated and shown in Figure 6, with mean R values of 0.53 for CGLS, 0.46 for MODIS, and 0.40 for QA4ECV. Similarly, all FAPAR agree well with soil moisture and better accuracy was observed in eastern Australia. Since eastern Australia is covered more by vegetated areas compared to western Australia, it might explain the better correlation between FAPAR and SPEI, as well as soil moisture over eastern Australia. It is noted that the weaker correlation of QA4ECV FAPAR with the other products may also partly be a consequence of the bias in the QA4ECV albedo data, which leads to non-systematic deviations in the QA4ECV FAPAR [74].

**Figure 7:**
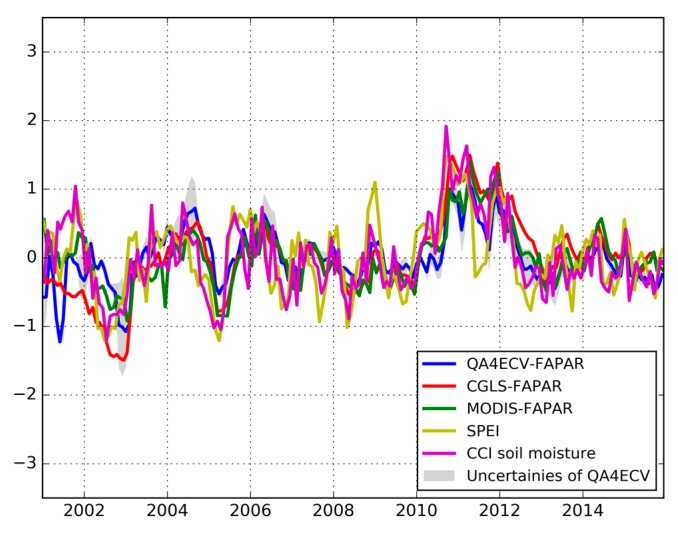
Temporal variation of area means of SPEI, standardized anomalies of analyzed FAPAR products, and the soil moisture anomaly for the period of 2001 to 2015. It is noted that the grey areas are the uncertainties of QA4ECV FAPAR.

In addition to the spatial comparison, the temporal correlation analysis between FAPAR anomalies and SPEI, and the soil moisture anomaly was investigated. Figure 7 shows the temporal variation of area mean values of all explored variables. It is noted that the uncertainties of QA4ECV is also shown in the figure, which is an advantage of the QA4ECV FAPAR compared to MODIS and CGLS FAPAR. We can see that the FAPAR anomalies show generally close agreement with SPEI and the soil moisture anomaly. For example, the FAPAR anomalies, SPEI, and the soil moisture anomaly present large negative values during the Millennium drought period (2001–2009) such as the years 2003 and 2005 [6]. However, all the variables show much higher values in 2011 than in the other years, which is due to the strong La Niña in 2010–2011 inducing heavy precipitation and large-scale flooding in Australia [75]. The results here imply that FAPAR can be used to monitor drought and that the recently developed QA4ECV has similar performance as MODIS and CGLS FAPAR for drought monitoring.

### 4.3. Spatial and Temporal Analysis for the 2005 Australia Drought

**Figure 8:**
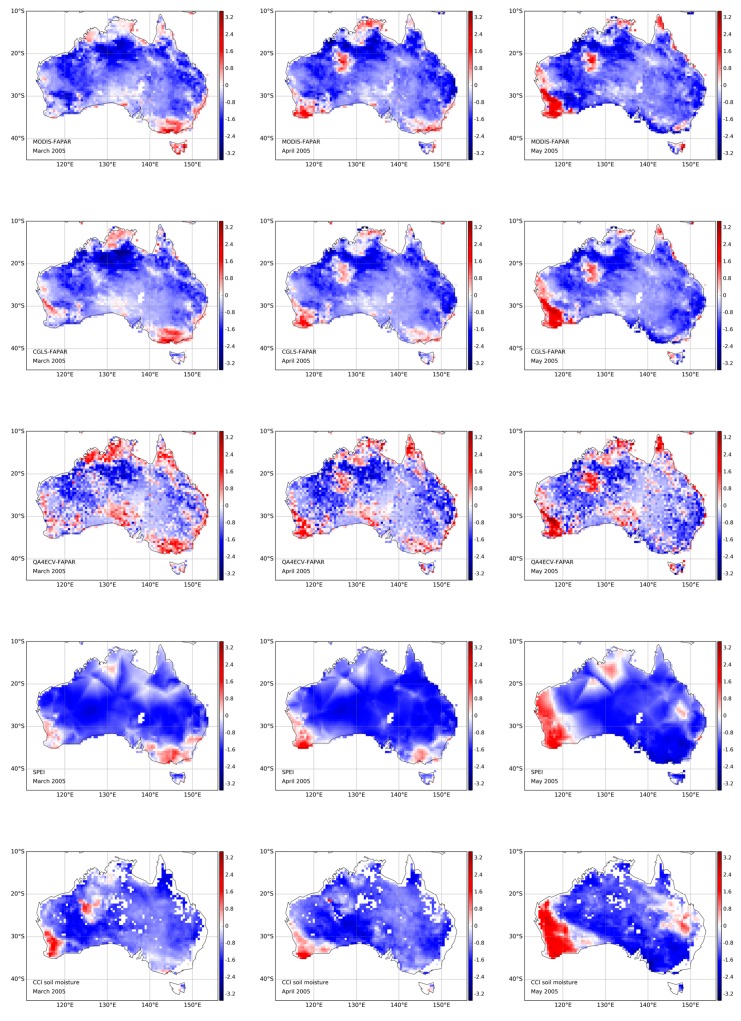
Temporal variation of spatially distributed FAPAR, CCI soil moisture standardized anomaly, and SPEI (**rows**) over Australia from March to May (**columns**) in 2005.

In this section, the temporal variations of spatial patterns of all the variables are explored during a typical drought year in 2005, that was within the Australian Millennium drought period (2001–2009) [6]. Figure 8 shows the latter from March to May in 2005. The negative values in blue represent the dry areas, while positive values in red identify wet areas. In general, all variables represent negative values for most of Australia from March to May, implying that the drought lasted without interruption for these areas. However, southwestern Australia changes from dry to wet, and southeastern Australia becomes drier from March to May. These variations were captured well by all FAPAR datasets considered here, as well as by SPEI and CCI soil moisture. Similar findings have been reported by Leblanc et al. [76] using the Gravity Recovery and Climate Experiment (GRACE) groundwater storage data. Similar patterns among all the FAPAR datasets further support the fitness of QA4ECV FAPAR in drought monitoring.

## 5. Discussion

This study has comprehensively explored the performance of different satellite-based FAPAR datasets for drought monitoring in Australia. The newly released QA4ECV FAPAR generally shows similar temporal and spatial patterns to MODIS and CGLS FAPAR. Since MODIS and CGLS FAPAR have been both validated and applied in previous studies, particularly for drought analyses such as [33,34,35], the agreement of QA4ECV FAPAR with these datasets suggests its potential for drought monitoring. The MODIS and CGLS FAPAR datasets have the advantage of higher spatial resolution, however they are limited to short temporal coverage; only since around the 2000s. The QA4ECV FAPAR accounts for the lack of longer time series and covers the time period from 1982 to 2016.

Regarding drought monitoring for agriculture and vegetation growth status, the FAPAR can serve as a proper proxy for greenness and vegetation health [35,77,78]. SPEI and soil moisture have been recognized as reliable tools for meteorological and hydrological drought monitoring. The inter-comparison between FAPAR, SPEI, and soil moisture can to some extent show to the ability of FAPAR for drought monitoring. The correlation analysis presented here shows that the FAPAR standardized anomaly generally agrees well with soil moisture standardized anomaly and SPEI. In particular, the high correlation appears in highly vegetated areas, which suggests the ability of FAPAR to be useful for agricultural drought monitoring [79]. These results are consistent with previous studies such as Ivits et al. [77], who reported good agreement between SPEI and FAPAR in the vegetation growing season. The similar performance given by MODIS, CGLS, and QA4ECV FAPAR endorses the ability of the latter dataset for drought monitoring in Australia. Another advantage of QA4ECV FAPAR is that the traceable uncertainty information is provided in the dataset, which are not available for other existing FAPAR datasets to the same extent.

Although the three FAPAR datasets considered here present many similarities, differences still exist. The reasons may be summarized as follows: (1) Different sensors and satellite platforms; (2) different retrieval methods and associated model-specific assumptions; (3) spectral responsivity differences. Specifically, Pickett-Heaps et al. [36] reported that the differences between satellite-based FAPAR datasets are largely due to different sensitivities in FAPAR to variations in vegetation cover. These inconsistencies are further related to changes in biome type and relevant to model-specific assumptions. Therefore, improvements of the retrieval methods and quantification of the uncertainties still need to be highlighted in future studies. The QA4ECV FAPAR was generated based on a quality assurance framework, which can provide traceable, reliable, and understandable uncertainty information from the propagation through the processing algorithms. The uncertainty information provides spatial and temporal information on the data quality to the user, which can be used in any application of the data, most notably data assimilation and data fusion.

**Figure 9:**
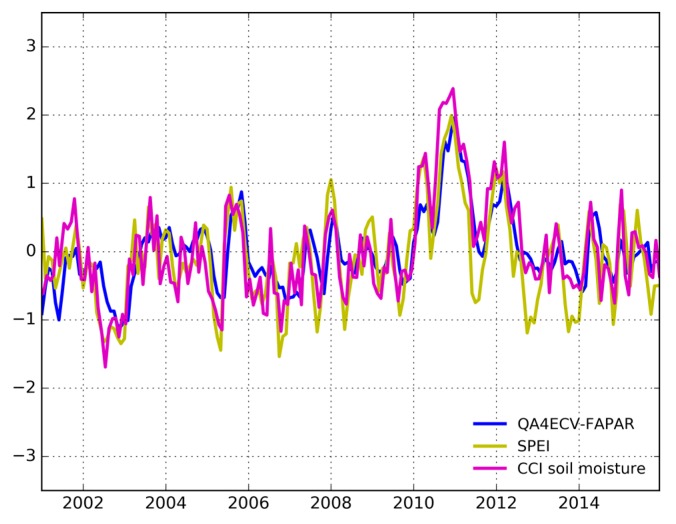
Time series of QA4ECV FAPAR, CCI soil moisture, and SPEI over Murray–Darling Basin, Australia.

It is noted that different drought indictors are based on different variables, which should provide complementary information on droughts. Take Murray–Darling Basin in southeast Australia as an example—the time series of different drought indicators are shown in Figure 9. It can be seen that SPEI, QA4ECV FAPAR, and CCI soil moisture show good consistency in seasonal variability but with large discrepancy in magnitude. In particular, large differences in magnitude are observed between SPEI and soil moisture, and FAPAR. Specifically, the SPEI shows greater water deficiency for years from 2010 to 2014 than both FAPAR and soil moisture, which show similar magnitude. This might be because SPEI is calculated based on precipitation and potential evapotranspiration, which can capture the loss of water well, while CCI soil moisture only measures the shallow surface of the soil. Therefore, the soil moisture drought tends to stabilize at low levels. Similar results have also been found by previous studies such as [6,70]. The better agreement in magnitude between soil moisture and FAPAR indicates that the shallow-rooted vegetation might decide the greenness in the Murray–Darling Basin. Zhao et al., [70] also found that NDVI has better agreement with soil moisture than the GRACE drought severity index. Therefore, different drought indicators should be used as complementary for drought monitoring and should not replace each other. For example, FAPAR, SPEI, and soil moisture have been selected and operationally used as the Combined Drought Indicator (CDI) for monitoring drought in the European Drought Observatory (EDO) within the Copernicus Emergency Management Service.

## 6. Conclusions

This study aimed to evaluate the newly developed QA4ECV FAPAR dataset for drought analysis in Australia. Firstly, the QA4ECV FAPAR was compared with MODIS and CGLS FAPAR datasets for a common time period from 2001 to 2015. Similar spatial patterns and high positive correlation were found among the datasets. In order to show to the ability of FAPAR for drought monitoring, the standardized anomaly of FAPAR was calculated and compared spatially and temporally with the drought index SPEI. Generally good agreement, particularly over highly vegetated areas, was found between FAPAR and SPEI. In addition, the same analysis was also conducted between the SPEI standardized anomaly and the CCI soil moisture standardized anomaly. Similar results were found between SPEI and CCI soil moisture, indicating the potential of FAPAR for drought monitoring. Similar performance was also found between different FAPAR datasets in the spatial and temporal analysis against SPEI and soil moisture, which suggests that the QA4ECV FAPAR dataset offers potential for drought monitoring. A case study of the 2005 Australia drought specifically shows the capability of FAPAR in monitoring the temporal and spatial variation of drought. Compared with MODIS and CGLS FAPAR, QA4ECV FAPAR has the advantages of being much longer-term, as well as providing traceable and reliable uncertainty information. In a future study, the QA4ECV FAPAR dataset will be further improved based on a reprocessed and bias-corrected QA4ECV albedo dataset.
